# Supplementary material for: Experimental demonstration of continuous quantum error correction
Source: Nat Commun. 2022 Apr 28;13:2307. doi: 10.1038/s41467-022-29906-0 (PMC9050892; doi:10.1038/s41467-022-29906-0)
Supplement: Supplementary file 1 — Supplementary Information [file 41467_2022_29906_MOESM1_ESM.pdf]

Supplementary Information: Experimental demonstration of continuous quantum error correction

William P. Livingston[1,2*], Machiel S. Blok[1,2,3], Emmanuel Flurin[4], Justin Dressel[5,6], Andrew N. Jordan[3,5], and Irfan Siddiqi[1,2]

[1]Department of Physics, University of California, Berkeley, CA 94720 USA
[2]Center for Quantum Coherent Science, University of California, Berkeley, California 94720, USA.
[3]Department of Physics and Astronomy, University of Rochester, Rochester, New York 14627, USA
[4]Université Paris-Saclay, CEA, CNRS, SPEC, 91191 Gif-sur-Yvette Cedex, France
[5]Institute for Quantum Studies, Chapman University, Orange, CA 92866, USA
[6]Schmid College of Science and Technology, Chapman University, Orange, CA 92866, USA

Supplementary Note 1: System parameters

We summarize qubit and resonator frequencies, as well as typical qubit lifetimes in the tables below. Each resonator has a Purcell filter centered at the same frequency.

	Q_0	Q_1	Q_2
Frequency (MHz)	5355	5182	5392
Anharmonicity (MHz)	307	310	310
T_1 (μs)	22	23	23
T_2^* (μs)	18	26	20
T_2^{echo} (μs)	31	31	35

Supplementary Table 1: Qubit parameters

	R_0	R_1
Frequency (MHz)	6314	6405
Linewidth, κ (kHz)	636	810
Dispersive shift, χ (MHz)	2.02	2.34
Quantum efficiency, η	0.62	0.56
Designed Purcell filter Q	30	30

Supplementary Table 2: Resonator parameters

Supplementary Note 2: Steady state dephasing

Here we derive relative dephasing rates for two qubits in a dispersive parity measurement using a classical analysis of the resonator steady states. The measurement dephasing rate is proportional to the distinguishability of resonator responses when the coupled qubits are in different eigenstates[1]. We set the probe frequency on resonance with the cavity when qubits are in the single-excitation subspace and assume that $\chi \gg \kappa$. We also assume the external cavity coupling is much larger than the internal cavity loss, so the cavity responds with the following scattering parameter:

$$S(f_0 := \chi \langle Z_0 + Z_1 \rangle) = \frac{-2f_0 + i\kappa}{-2f_0 - i\kappa} \tag{S.1}$$

Odd parity states are perfectly indistinguishable. The distinguishability between states of opposite parity is

$$D_{01,00} = |S(0) - S(2\chi)|^2 = \left| (-1) - \frac{-4\chi + i\kappa}{-4\chi - i\kappa} \right|^2 \approx 4 \tag{S.2}$$

With $z \equiv 4\chi + i\kappa$, the distinguishability between the two even parity states is:

$$\begin{aligned} D_{11,00} = |S(-2\chi) - S(2\chi)|^2 &= \left| \frac{4\chi + i\kappa}{4\chi - i\kappa} - \frac{-4\chi + i\kappa}{-4\chi - i\kappa} \right|^2 \\ &= \left| \frac{z}{\bar{z}} - \frac{\bar{z}}{z} \right|^2 = \left| \frac{z^2 - \bar{z}^2}{|z|^2} \right|^2 = \left| \frac{(z+\bar{z})(z-\bar{z})}{|z|^2} \right|^2 \\ &= \left(\frac{(8\chi)(2\kappa)}{16\chi^2 + \kappa^2} \right)^2 \approx \left(\frac{\kappa}{\chi} \right)^2 \end{aligned} \tag{S.3}$$

From these equations, we get the following relative dephasing (Γ) and measurement rates (Γ^m) between states of different parity and states of even parity:

$$\frac{\Gamma_{01,00}}{\Gamma_{11,00}} = \frac{\Gamma^m_{01,00}}{\Gamma^m_{11,00}} \approx \frac{4\chi^2}{\kappa^2} \tag{S.4}$$

Supplementary Note 3: Dynamic Dephasing

When the resonator is not at steady state, one can have significantly increased dephasing rates after a parity flip. Here we will consider the effect of a bit flip error taking an odd parity qubit state to an even parity state while the parity measurement is on. In this case, the measurement tone is on resonance with the cavity and the cavity field will initially be in a steady state α_0. When the qubit parity is flipped from odd to even, the cavity evolves as two copies, one for each even parity basis state (α_{00} and α_{11}). As a simplifying approximation, we assume the measurement tone is turned off at the moment the parity changes as to capture just the transient dynamics. There are two equivalent methods[1] to calculate the net dephasing ζ. The first can be obtained by integrating the

rate at which information leaves the cavity, $\Gamma_\phi^m = \frac{\kappa}{2}|\alpha_{00} - \alpha_{11}|^2$. The second can be obtained by integrating the rate at which the cavity dephases the qubit, $\Gamma_\phi = 4\chi\,\mathrm{Im}[\alpha_{00}\alpha_{11}^*]$, with 4χ being the frequency difference between the $|00\rangle$ resonance and the $|11\rangle$ resonance. Here we use the second method to simplify the calculation. We work in the rotating frame of the odd-parity resonance and define $k \equiv \kappa/2 - 2i\chi$ to get two cavity equations, one associated with each basis state:

$$\begin{aligned}\dot{\alpha}_{00} &= \left(2\chi i - \frac{\kappa}{2}\right)\alpha_{00} \\ \dot{\alpha}_{11} &= \left(-2\chi i - \frac{\kappa}{2}\right)\alpha_{11}\end{aligned} \tag{S.5}$$

$$\begin{aligned}\alpha_{00}(t) &= \alpha_0 e^{-kt} \\ \alpha_{11}(t) &= \alpha_0 e^{-\bar{k}t}\end{aligned} \tag{S.6}$$

$$\begin{aligned}\zeta = \int_0^\infty 4\chi\,\mathrm{Im}\left[\alpha_{00}\alpha_{11}^*\right]dt &= 4\chi\,\mathrm{Im}\left[\int_0^\infty \alpha_{00}\alpha_{11}^* dt\right] \\ &= |\alpha_0|^2\, 4\chi\,\mathrm{Im}\left[\int_0^\infty e^{-2kt}dt\right] \\ &= |\alpha_0|^2\, 4\chi\,\mathrm{Im}\left[\frac{1}{2k}\right] = |\alpha_0|^2\, 4\chi\,\mathrm{Im}\left[\frac{2\bar{k}}{|2k|^2}\right] \\ &= |\alpha_0|^2 \frac{16\chi^2}{\kappa^2 + 16\chi^2} \approx |\alpha_0|^2\end{aligned} \tag{S.7}$$

Therefore, the magnitude of the final coherence between $|00\rangle$ and $|11\rangle$, $|\rho^f_{00,11}|$, will be dephased from the initial coherence between $|01\rangle$ and $|10\rangle$, $|\rho^i_{01,10}|$:

$$\left|\rho^f_{00,11}\right| = e^{-\zeta}\left|\rho^i_{01,10}\right| = e^{-|\alpha_0|^2}\left|\rho^i_{01,10}\right| \tag{S.8}$$

Supplementary Method 1: Tomographic reconstruction

We use the parity resonators to perform qubit tomography. However, due to the nature of the parity condition, not all states are distinguishable by this measurement. To perform tomography, we use single qubit pulses to map each three-qubit Pauli eigenstate to $|000\rangle$ and then measure both resonators on their respective $|00\rangle$ resonance. We then measure the probability that full qubit system is in the ground state, which corresponds to reading out both resonators as 0. We additionally include data into the tomography analysis if one of the resonators reads out 1 and the other reads out 0, since we know the final state to be in either $|100\rangle$ or $|001\rangle$ depending on which resonator reads 1. Using this information, we construct partial Pauli expectation values such as $\langle X^+Y^-I\rangle$,

with P^+, P^- being the plus and minus projectors for a particular Pauli $P \in \{X, Y, Z\}$ such that $P = P^+ - P^-$. We then apply readout correction on these probabilities to mitigate the effects of readout infidelity. From this corrected data taken over many tomographic sequences, we can reconstruct full Pauli expectation values such as $\langle XYI \rangle$. When reconstructing logical coherences, we only measure in the X and Y bases. When reconstructing populations, we only measure in the Z basis.

Supplementary Method 2: Ramsey heralding

Qubits 0 and 2 demonstrate a strong temporal bistability in qubit frequency, with a splitting of about 80 kHz and a typical switching time on the order of .1–10 s. When taking data to reconstruct logical coherences, we include five extra sequences in our AWG sequence table, each consisting of five repeated restless Ramsey measurements with free precession times of $6\,\mu\text{s}$. With a typical initial sequence length of 64 and a repetition rate of $100\,\mu\text{s}$, the qubit's frequency state is sampled every 7 ms, allowing us to herald data runs to only include data from runs when the qubits have a particular frequency.

Supplementary Method 3: Photon number calibration

To calibrate the number of photons in our resonator at steady state, we first calibrate the quantum efficiency of each chain by measuring the total dephasing and the SNR of a variable amplitude readout pulse[2]. We then measure the steady state measurement rate of an odd versus even parity state by measuring steady state SNR increase between an even and odd state. Using the relation between measurement rate and measurement induced dephasing[1], we find $\Gamma_m = 2\eta\Gamma_\phi^m = \kappa\eta|\alpha_{00} - \alpha_{01}|^2 \approx \kappa\eta\bar{n}$. Here, Γ_m is the measurement rate and Γ_ϕ^m is the measurement induced dephasing rate. We also have approximated the even parity state as having a much lower average photon number than the odd parity state, which is the case when measuring on the odd parity resonance with $\chi \gg \kappa$. Using this method we determine the steady state photon numbers in R_0 and R_1 to be .7 and .6 respectively.

Supplementary Figures

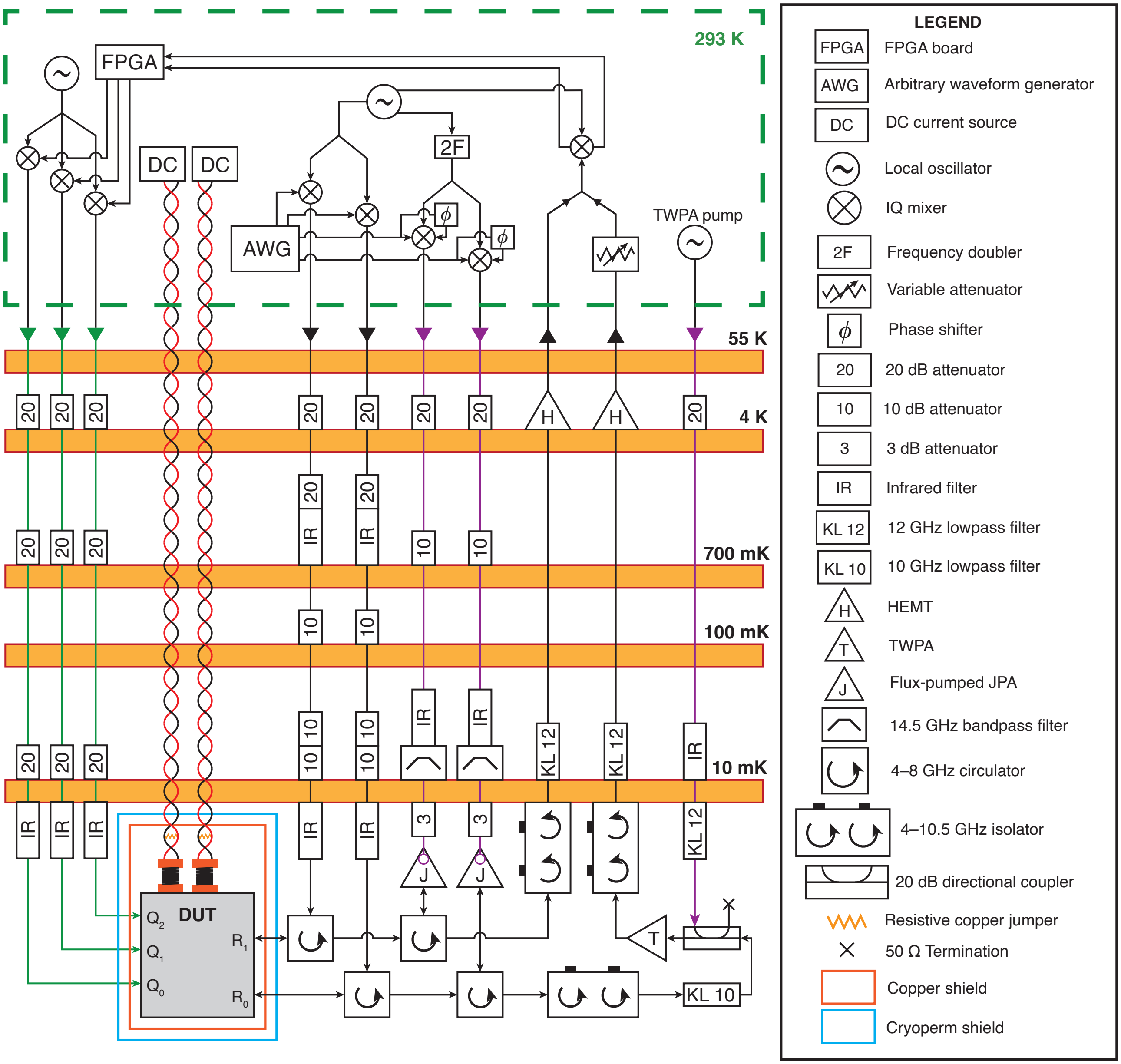

Supplementary Figure 1: **Cryogenic wiring diagram.** The Josephson parametric amplifiers (JPAs) operate in reflection, and additionally have off chip coils not shown. The JPAs also provide narrow-band gain, so when the readout chains are combined at room temperature, the combined noise at each cavity frequency is dominated by the noise amplified by that cavity's JPA. Each superconducting coil has its leads connected by a small piece of copper wire on the sample box, forming a low frequency ($< 1\,\mathrm{Hz}$) RL filter with the coil. The room temperature wiring is also shown, but with linear elements (attenuators, amplifiers, filters, isolators) removed.

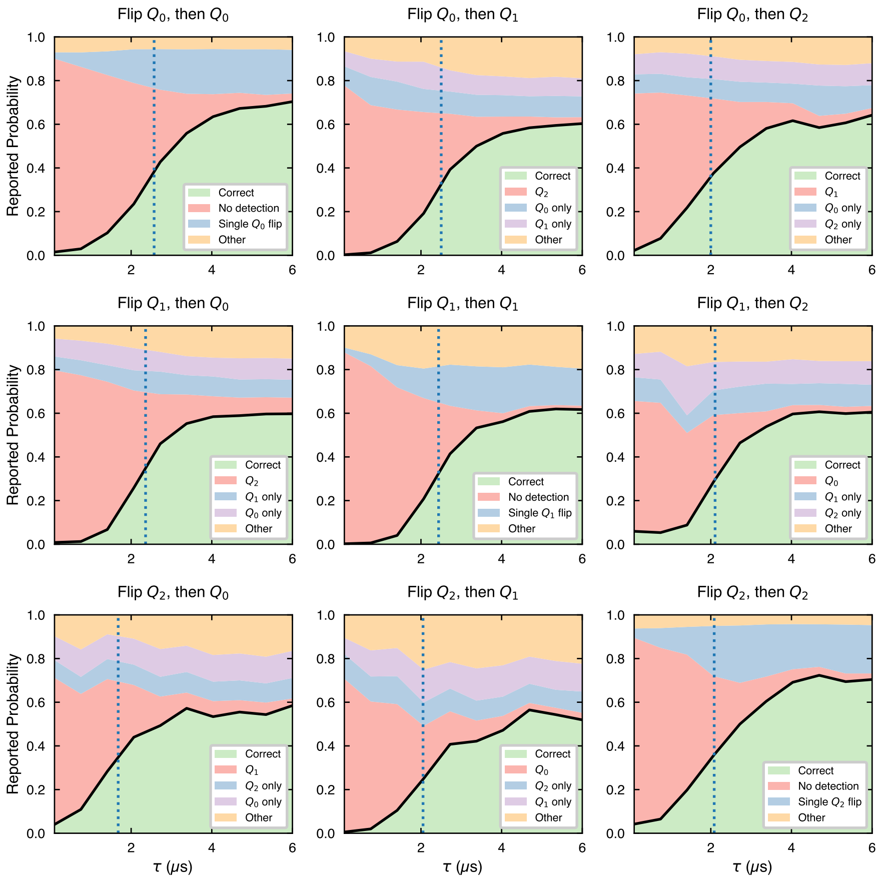

Supplementary Figure 2: **Extended data plot for Figure 3b.** Pairs of qubits are flipped with varying time between flips. When different qubits are flipped, the red region represents the controller detecting a flip on the third qubit, resulting in a logical error. When the same qubit is flipped twice, the red region represents the probability of the controller not detecting a flip (which is not a logical error). Blue and purple regions represent the probability of a single flip being detected. The orange region represents the probability of the controller detecting some other sequence of flips. Dotted lines represent the dead time, when the red probability matches the green probability.

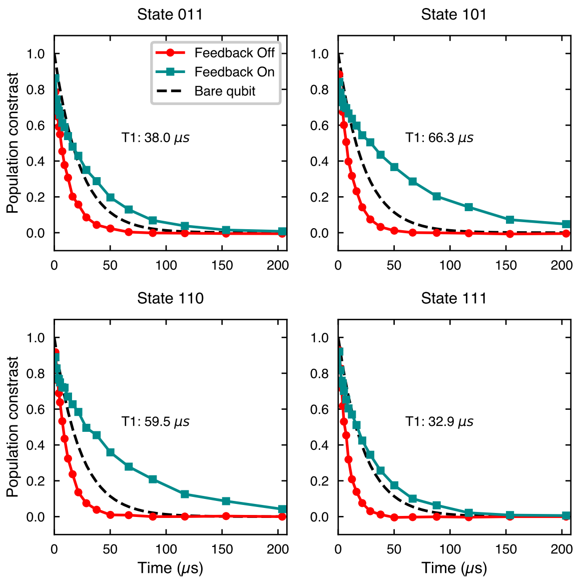

Supplementary Figure 3: **Extended data plot for Figure 3c.** Effective T_1 for all four definite parity subspaces are measured and fit to an exponential. Red traces are taken with feedback off. Blue traces are taken with feedback on. The black dotted line represents the lifetime of a bare qubit (24 μs).

Supplementary References

1. Gambetta, J. *et al.* Quantum trajectory approach to circuit qed: Quantum jumps and the zeno effect. *Phys. Rev. A* **77**, 012112 (2008). URL https://link.aps.org/doi/10.1103/PhysRevA.77.012112.

2. Bultink, C. C. *et al.* General method for extracting the quantum efficiency of dispersive qubit readout in circuit qed. *Applied Physics Letters* **112**, 092601 (2018). URL https://doi.org/10.1063/1.5015954. https://doi.org/10.1063/1.5015954.
